# Frequent implementation of interventions may increase HIV infections among MSM in China

**DOI:** 10.1038/s41598-017-18743-7

**Published:** 2018-01-11

**Authors:** Xiaodan Sun, Yanni Xiao, Zhihang Peng, Ning Wang

**Affiliations:** 1 Department of Applied Mathematics, Xi’an Jiaotong University, Xi’an, 710049 China; 2 Department of Epidemiology and Biostatistics, Nanjing Medical University, Nanjing, 210029 China; 3 National Center for AIDS/STD Prevention and Control, Chinese Center for Disease Control and Prevention, Beijing, 102206 China

## Abstract

Intervention measures among men who have sex with men (MSM) are usually designed to reduce the frequency of high risk behaviors (within-community level), but unfortunately may change the contact network and consequently increase the opportunity for them to have sex with new partners (between-community level). A multi-community periodic model on complex network is proposed to study the two-side effects of interventions on HIV transmission among MSM in China, in which the wanning process of the impacts of interventions are modelled. The basic reproduction number for the multi-community periodic system is defined and calculated numerically. Based on the number of annual reported HIV/AIDS cases among MSM in China, the unknown parameters are estimated by using MCMC method and the basic reproduction number is estimated as 3.56 (95%CI [3.556, 3.568]). Our results show that strong randomness of the community-connection networks leads to more new infections and more HIV/AIDS cases. Moreover, main conclusion indicates that implementation of interventions may induce more new infections, depending on relative level of between- and within-community impacts, and the frequency of implementation of interventions. The findings can help to guide the policy maker to choose the appropriate intervention measures, and to implement the interventions with proper frequency.

## Introduction

Human immunodeficiency virus (HIV) spreads quickly in China after the first AIDS case was identified in 1985^1^. In recent years, the prevalence of HIV among men who have sex with men (MSM) has increased significantly in China since 2005. The proportion of new reported cases attributed by MSM from 2005 to 2014 increases from 0.3%, to 25.8% respectively^2–4^. Compared with that among other high risk population, HIV infection among MSM is featured by higher infection rate per high risk behavior with unprotection^5,6^, lower condom usage rate^7^ and more sex partners^8,9^. It is essential to develop some novel strategies to effectively curb the quick spread of HIV infections in MSM groups.

To prevent the epidemic from further spread, a lot of prevention and control strategies, including condom promotion, counseling and test, peer education and propaganda education, have been implemented at a national level in recent years^10–15^. Besides the Chinese government, community-based organizations(CBOS) committed to the control of HIV have also been substantially strengthened with the support of international aid^16,17^. Since MSM groups bear low levels of HIV knowledge, perceived risk, and high rates of sexually transmitted diseases^18–20^, the development of novel interventions, including providing telephone counseling^21^, supervising the MSM population by using QQ group^22^ and promoting the CBOS to engage in HIV prevention and control^23^, have been called for by several researchers^14,15^. Intervention measures are designed to reduce the high risk behaviors by increasing condom usage rate, reducing sex partners and etc. However, some recently proposed interventions, such as group counseling, workshops, community-building empowerment activities and CBOS, may change the connection network among MSMs, and hence increase the chance for gay men to know new partners. When Wu *et al*.^8^ evaluated the effects of intervention on risk behaviours among 244 MSMs in colleges, they found that the mean number of sex partners per gay men increased from 3 to 4 after the implementation of interventions. Hence, intervention strategies have two-side effects on behavior changes. On one hand, interventions increase the rate of protection such as condom use, which decreases transmission probability, on the other hand, they may bring more new partners, which increases the contact rates. There are many studies examining the effects of intervention measures on the behavior changes of gay men^8,24–28^, in which impact of interventions has been modelled positively with a constant reduction factor. Many challenges remain in quantifying and evaluating the two-side effects of these intervention strategies among MSMs on the control of HIV infection and fall within the scope of this study.

The purpose of this study is to quantify and examine the two-side impacts of interventions, investigate their comprehensive effects on HIV infections among MSMs through a mathematical modeling framework and then suggest the feasible strategies to curb HIV infections. To describe new links possibly formulated among various MSM communities once interventions are implemented, we employ the meta-population modelling idea^29–32^ and propose a multi-patchy model to examine the two-side effect of the interventions among MSM groups. Here one community corresponds to one patch, thus we name our model as multi-community model. In the research of infectious disease, meta-population models are widely used to either describe the population dispersal from one patch to another^29,30,33–35^, or account the cross transmission^31,36–38^, where infected individuals in one patch can infect individuals in other patches. The existing work mainly treats effect of intervention measures as constant on reducing transmission dynamics^32,39,40^, which fails to consider the side-effect of inventions as mentioned above, or ignores wanning process of the impacts of interventions. In fact, the impacts of interventions on behavior usually wan with time, which will firstly be modeled in this study. We then focus on the following essential issues: how often and in what scale the interventions should be implemented in terms of reducing new infection? What kind of interventions should be implemented? How does network structure between various communities affect the transmission dynamics or new HIV infections?

## Methods

### Model formulations

As stated in introduction, an intervention may have two-side impacts on behavior changes: increasing the number of new sex partners from other communities, which is called as the between-community impact, or enhancing protection per high risk behavior, which is thought to be the within-community impact. Before the behavioral interventions are implemented, we assume *n* communities are isolated from each other. That is, individuals in different communities have no contacts, thus no cross-infection from different communities happens. However, with interventions, individuals in different communities may begin to ‘know’ each other, thus infected individuals can infect not only individuals from the same community but also individuals from the connected communities. In the following, we initially formulate the model in a single community and then extend it to a full multi-community model on a network structure.

#### The single-community model

We initially consider the key epidemiological properties of the HIV/AIDS epidemic without intervention measures. In each community *i*, we adopt the model which is formulated by Xiao *et al*.^32^ based on characteristics of nationwide database. The individuals in community *i* are divided into 4 classes: high-risk susceptibles (*S*), HIV infected individuals who are not yet diagnosed (*I*), diagnosed HIV-positive individuals(*D*_*I*_) and diagnosed AIDS patients(*D*_*A*_). The model equations are

1 $$\{\begin{array}{rcl}\frac{d{S}_{i}}{dt} & = & {U}_{i}-{\beta }_{ii}{S}_{i}\frac{{I}_{i}+\varrho {D}_{{I}_{i}}+\varepsilon {D}_{{A}_{i}}}{{N}_{i}}-({\mu }_{i}+d){S}_{i},\\ \frac{d{I}_{i}}{dt} & = & {\beta }_{ii}{S}_{i}\frac{{I}_{i}+\varrho {D}_{{I}_{i}}+\varepsilon {D}_{{A}_{i}}}{{N}_{i}}-(d+{\delta }_{i}){I}_{i},\\ \frac{d{D}_{{I}_{i}}}{dt} & = & \rho {\delta }_{i}{I}_{i}-(\xi +d+{\alpha }_{I}){D}_{{I}_{i}},\\ \frac{d{D}_{{A}_{i}}}{dt} & = & \mathrm{(1}-\rho ){\delta }_{i}{I}_{i}+\xi {D}_{{I}_{i}}-(d+{\alpha }_{A}){D}_{{A}_{i}}\mathrm{.}\end{array}$$

where ${N}_{i}={S}_{i}+{I}_{i}+{D}_{{I}_{i}}+{D}_{{A}_{i}}$ denotes high-risk population size of community *i*. Individuals enter into the susceptible class at a rate *U*_*i*_, exit the high-risk group at a constant rate *μ*_*i*_, and become infected at a rate ${\beta }_{ii}(I+\varrho {D}_{{I}_{i}}+\varepsilon {D}_{{A}_{i}}){S}_{i}/{N}_{i}$. Here, ${\beta }_{ii}={c}_{i}{\beta }_{ii}^{0}$, where ${\beta }_{ii}^{0}$ denotes the transmission probability per high-risk behavior, *c*_*i*_ is the contact rate. The natural death rate for each class is *d*. Parameters $\varrho$ and *ε* are the modification factors accounting for varying levels of the activity and infectiousness of the diagnosed HIV-positive individuals and the AIDS patients; *δ*_*i*_ represents the diagnosis rate; *ρ* is the proportion of diagnosed individuals who have not yet progressed to AIDS; *ξ* is the rate of progression from HIV diagnosis to the AIDS class; *α*_*I*_ and *α*_*A*_ denote the disease related death rates for the diagnosed HIV-positive individuals and those living with AIDS, respectively.

#### The multi-community model on contact networks

Once interventions are implemented, the incidence rate within communities may decrease, while the incidence rate among connected communities may increase due to two-side effects of interventions. Let *v*_*ii*_(*t*) be the rate of protection, *σ*_*i*_(*t*) be the additional rate of exiting high-risk group of community *i* because of the interventions. Let *v*_*ij*_(*t*) be additional rate of cross-infection among communities *i* and *j* due to interventions. For convenience, *v*_*ii*_(*t*) and *v*_*ij*_(*t*) are called as within- and between-community impact functions of interventions. To model the waning of impacts of interventions with time after implementation of interventions, we assume that the impact of intervention linearly decreases with a constant rate from its maximum value. Thus, both *v*_*ii*_(*t*) and *v*_*ij*_(*t*) are dynamic rather than constants and have the following equations.

2 $$\{\begin{array}{llll}\frac{d{v}_{ii}(t)}{dt} & = & -{r}_{ii}^{v}{v}_{ii}(t), & t\ne k{T}_{l}\\ \frac{d{v}_{ij}(t)}{dt} & = & -{r}_{ij}^{v}{v}_{ij}(t), & t\ne k{T}_{l},\\ \frac{d{\sigma }_{i}(t)}{dt} & = & -{r}_{i}^{\sigma }{\sigma }_{i}(t), & t\ne k{T}_{l},\\ {v}_{ii}(k{T}_{l}^{+}) & = & {v}_{ii}^{m}, & t=k{T}_{l},\\ {v}_{ij}(k{T}_{l}^{+}) & = & {v}_{ij}^{m}, & t=k{T}_{l},\\ {\sigma }_{i}(k{T}_{l}^{+}) & = & {\sigma }_{i}^{m}, & t=k{T}_{l}\mathrm{.}\end{array}$$

Here ${v}_{ii}^{m},{v}_{ij}^{m}$ and ${\sigma }_{i}^{m}$ are the maximum value of *v*_*ii*_(*t*), *v*_*ij*_(*t*) and *σ*_*i*_(*t*), and the constant wanning rates are ${r}_{ii}^{v},{r}_{ij}^{v}$ and ${r}_{i}^{\sigma }$, respectively. Note that intervention measures are used to be implemented impulsively instead of continuously, for simplicity, we assume that interventions are implemented periodically with the implementation period of *T*_*l*_.

Here, we suppose that interventions can cover *n* communities. Communities are connected through network structures after implementation of interventions. The connection network is described by an adjacent matrix *M* = (*m*_*ij*_)_*i*,*j*_ = 1, …, *n* with elements of 0 and 1, and *m*_*ij*_ = 1 if and only if community *i* is connected to community *j*. Thus, we have

3 $$\{\begin{array}{rcl}\frac{d{S}_{i}}{dt} & = & {U}_{i}-{\beta }_{ii}\mathrm{(1}-{v}_{ii}(t)){S}_{i}\frac{{I}_{i}+\varrho {D}_{{I}_{i}}+\varepsilon {D}_{{A}_{i}}}{{N}_{i}}\\  &  & -{\sum }_{j\ne i}{\beta }_{ij}{m}_{ij}{v}_{ij}(t){S}_{i}\frac{{I}_{j}+\varrho {D}_{{I}_{j}}+\varepsilon {D}_{{A}_{j}}}{{N}_{j}}-({\mu }_{i}+{\sigma }_{i}(t)+d){S}_{i},\\ \frac{d{I}_{i}}{dt} & = & {\beta }_{ii}\mathrm{(1}-{v}_{ii}(t)){S}_{i}\frac{{I}_{i}+\varrho {D}_{{I}_{i}}+\varepsilon {D}_{{A}_{i}}}{{N}_{i}}\\  &  & +{\sum }_{j\ne i}{\beta }_{ij}{m}_{ij}{v}_{ij}(t){S}_{i}\frac{{I}_{j}+\varrho {D}_{{I}_{j}}+\varepsilon {D}_{{A}_{j}}}{{N}_{j}}-(d+{\delta }_{i}){I}_{i},\\ \frac{d{D}_{{I}_{i}}}{dt} & = & \rho {\delta }_{i}{I}_{i}-(\xi +d+{\alpha }_{I}){D}_{{I}_{i}},\\ \frac{d{D}_{{A}_{i}}}{dt} & = & \mathrm{(1}-\rho ){\delta }_{i}{I}_{i}+\xi {D}_{{I}_{i}}-(d+{\alpha }_{A}){D}_{{A}_{i}}\mathrm{.}\end{array}$$

Equations (3) together with equations (2) are called as multi-community model on contact network. *It is supposed that the average maximum within-community and between-community high risk behaviors for each individual are independent of the number of communities. That is, both* $\frac{1}{n}{\sum }_{i=1}^{n}{v}_{ii}^{m}$ *and* $\frac{1}{n}{\sum }_{i=1}^{n}{\sum }_{i\ne j}{m}_{ij}{v}_{ij}^{m}$ *are unrelated to n. Thus, no matter what the number of communities covered by the interventions, just after interventions are implemented, each individual has on average the same probability to have between-community high risk behaviors. Let the between-community impacts of interventions v*_*ij*_(*t*) *satisfy that* ${v}_{ij}^{m}={v}_{between}^{\ast }/k$ *, where k is the mean number of neighbour communities for each community and constant* ${v}_{between}^{\ast }$ *denotes the average maximum between-community impacts of interventions. Then, within a period of implementation of intervention, we have*

$$\begin{array}{rcl}\frac{1}{n}\sum _{i=1}^{n}\sum _{i\ne j}{m}_{ij}{v}_{ij}^{m} & = & \frac{1}{n}\sum _{i=1}^{n}\sum _{i\ne j}{m}_{ij}{v}_{between}^{\ast }/k\\  & \approx  & {v}_{between}^{\ast }\end{array}$$

*If we further suppose that* ${r}_{ii}^{v}={r}_{w}^{v},\,{r}_{ij}^{v}={r}_{b}^{v},\,i\ne j,i,j=1\,\cdots ,n$, *where* ${r}_{w}^{v}$ *and* ${r}_{b}^{v}$ *are constants, we have* $\frac{1}{n}{\sum }_{i=1}^{n}{v}_{ii}(t)={v}_{ii}^{m}{e}^{-{r}_{ii}^{v}(t-k{T}_{l})}$ *and* $\frac{1}{n}{\sum }_{i=1}^{n}{\sum }_{i\ne j}{m}_{ij}{v}_{ij}(t)\approx {v}_{between}^{\ast }{e}^{-{r}_{ij}^{v}(t-k{T}_{l})}$. *Thus*, *the average within-community and between-community high risk behaviors for each individual are always independent of the number of communities*.

It is not difficult to get that any solution of (3) with nonnegative initial value is nonnegative. Then, the set *G* defined by

$$\begin{array}{rcl}G & = & \{({S}_{1},{I}_{1},{D}_{I1},\cdots ,{S}_{n},{I}_{n},{D}_{In},{D}_{In})\in {R}_{+}^{4n}|0\le {\sum }_{i=1}^{n}({S}_{i}+{I}_{i}+{D}_{Ii}\\  &  & +{D}_{Ai})\le \sum _{i=1}^{n}\,\frac{{U}_{1}}{{\mu }_{i}+{\sigma }_{i}^{l}+d} < +\infty ,\,\,{\rm{where}},\,\,{\sigma }_{i}^{l}=\mathop{{\rm{\min }}}\limits_{t\in \mathrm{[0,}{T}_{i}]}{\sigma }_{i}(t)\}\mathrm{.}\end{array}$$

is a positively invariant set for system (3).

### Data and parameter estimation

**Figure 1:**
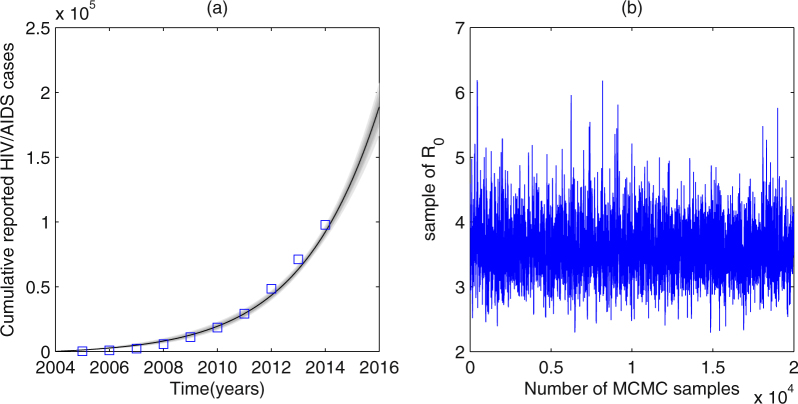
Plots of data fitted results. (**a**) The number of accumulated annual reported HIV/AIDS cases. (**b**) MCMC plots for *R*_0_. Squares denote the real data. Areas from light to dark denote the 50%, 90%, 95% and 99% predictive probability limits due to parameter uncertainties.

Based on the current surveillance system of China, the number of annual reported HIV-positive cases and AIDS patients among MSM from the year 1985 to 2009 in mainland China can be obtained. The number of annual reported HIV/AIDS cases from year 2010 to 2013 can be obtained in the website of ‘The Data-center of China Public Health Science’^41^, and the proportions attributed by MSM from year 2006 to 2014 as well as the number of new reported HIV/AIDS cases in 2014 are illustrated in the 2015 China AIDS response progress report^2^. By simple calculations, we can get the number of annual accumulated reported HIV/AIDS cases among MSM from year 2010 to 2014. In this article, the data from the year 2005 to year 2014, shown in Fig. 1(a), are used since the consistent surveillance and test policy have been implemented since then^32^. Only gay men between age 15 and 64 are considered according to the actual situation of MSM population, thus *μ*_*i*_ = 0.02. The value of several parameters can be obtained from references or by simple calculations based on the database^32,39,42,43^, which are described in Table 1.

**Table 1:** Parameter values.

Parameters	Definition	Mean value	Std	Source
*U*_*i*_	Recruitment rate in patch *i*	100	—	assumed
*μ*_*i*_	Exit rate in patch *i*	0.02 yr^−1^	—	see text
*d*	Natural death rate	0.0149 yr^−1^	—	^42^
*β*_*ii*_	Transmission rate within patch *i*	0.5320	0.0036	MCMC
*β*_*ij*_	Transmission rate in patch *i* caused by infected individual in group *j*	≥0	—	see text
ϱ	Modification factor in transmission coefficient of diagnosed HIV–positive individuals	0.1100	0.0058	MCMC
*ε*	Modification factor in transmission coefficient of AIDS patients	0	—	^43^
*δ*_*i*_	Diagnosis rates in patch *i*	0.1437 yr^−1^	0.0199	MCMC
*ρ*	Proportion of HIV–positive individuals when diagnosed	0.88	—	^32,39^
*ξ*	Rate of progression to AIDS	0.116 yr^−1^	—	^39^
*α*_*I*_	Disease–related death rate for diagnosed HIV–positive individuals	0.172 yr^−1^	—	^39^
*α*_*A*_	Disease-related death rate for AIDS patients	0.318 yr^−1^	—	^39^
*k*	Mean number of neighbour communities for each community	> = 0	—	see text
*v*_*ii*_(*t*)	Within–community (community i) impact function of interventions	0–1	—	see text
*v*_*ij*_(*t*)	Between–community (communities i and j) impact function of interventions	0–1	—	see text
*σ*_*i*_(*t*)	High risk behavior give up function after interventions	0–1	—	see text
${v}_{ii}^{m}$	Maximum within–community (community i) impact	0–1	—	see text
${v}_{between}^{\ast }$	Average maximum between–community impacts for each community	0–1	—	see text
${v}_{ij}^{m}$	Maximum between–community (communities i and j) impact	${v}_{between}^{\ast }/k$	—	see text
${r}_{ii}^{v}$	Wanning rate of within–community (community i) impact	>0	—	see text
${r}_{ij}^{v}$	Wanning rate of between–community (communities i and j) impact	>0	—	see text
${\sigma }_{i}^{m}$	Maximum high risk behavior give up rate for community i after interventions	0–1	—	see text
${r}_{i}^{\sigma }$	Wanning rate of the high risk behavior give up rate of community i after interventions	>0	—	see text
*T*_*l*_	Implementation period for interventions	>0	—	see text

Lack of the data associated with interventions, we estimate the unknown parameters involved in single-community system (1). Since the number of susceptibles is large enough compared with the number of HIV/AIDS cases, we simply assume *S*_*i*_/*N*_*i*_ = 1^32,39^. Then main epidemiological parameters involved in the last three equations of the model (1) are estimated by using MCMC method^44^. Here, Metropolis- Hastings (M-H) algorithm is employed, and the algorithm runs for 40000 iterations with a burn-in of 20000 iterations. Let *θ* = (*β*_*ii*_, ϱ, *δ*_*i*_), and denote $f(\theta ,t)={\int }_{2004}^{t}{\delta }_{i}{I}_{i}(\tau )d\tau$ and *P*(*t*) as the numerical and real accumulative annual reported HIV/AIDS cases (*t* = 2005, …, 2014), the MCMC algorithm will minimize $s={\sum }_{t=2005}^{t=2014}{(P(t)-f(\theta ,t))}^{2}$.

## Results

### Basic reproduction numbers

Firstly, it is not difficult to prove that the system (2–3) has a *T*_*l*_− periodic disease-free periodic solution ${x}^{\ast }(t)=({\hat{S}}_{1}(t),0,0,0,\cdots ,{\hat{S}}_{i}(t),0,0,0,\cdots ,{\hat{S}}_{n}(t),0,0,\mathrm{0)}$ with ${\hat{S}}_{i}(t)$ shown in the supporting information. Using the theory proposed by Wang and Zhao^45^ we can define a linear operator $L:{C}_{{T}_{l}}\to {C}_{{T}_{l}}$ as follows (See details in the supporting information - SI).

$$(L\psi )\,(t)={\int }_{0}^{\infty }Y(t,t-a)\,F(t-a)\,\psi (t-a)da,\,\forall t\in R,\,\psi \in {C}_{{T}_{l}}\mathrm{.}$$

Where *ψ*(*s*) (*T*_*l*_ periodic in s) is the initial distribution of infectious individuals. The distribution of new infections produced by the infected individuals who were introduced at time *s* is *F*(*s*)*ψ*(*s*). And the distribution of infected individual who are infected at time *s* and still infectious at time *t* can be denoted by *Y*(*t*, *s*)*F*(*s*)*ψ*(*s*). The detailed formulas are given in the supporting information. Here function *F*(*t*) is piecewise continuous, with which we can also define the next generation operator^29,46^ and obtain the basic reproduction number. The basic reproduction number^47^ of system (2–3) can be defined as the spectral of the operator *L*, that is,

$${R}_{0}\,:\,=\,\rho (L\mathrm{).}$$

Let *W*(*t*, *λ*) denote the monodromy matrix of the *T*_*l*_− periodic system with parameter *λ* ∈ (0, ∞), which gives:

$$\frac{d\omega }{dt}=(-V(t)+\frac{1}{\lambda }F(t))\omega ,t\in R\mathrm{.}$$

As *F*(*t*) is nonnegative and −*V*(*t*) is cooperative, we have *r*(*W*(*T*_*l*_, *λ*)), representing the spectral radius of matrix *W*(*T*_*l*_, *λ*), is continuous and non-increasing for *λ* ∈ (0, ∞), and ${\mathrm{lim}}_{\lambda \to \infty }\,r(W({T}_{l},\lambda )) < 1$. Then, we can calculate the basic reproduction number numerically by finding the positive solution *λ*_0_ of *r*(*W*(*T*_*l*_, *λ*)) = 1 on the basis of the Lemma proved by Wang and Zhao^45^ (or see details in the SI).

By fitting the reduced model (1) with *S*_*i*_/*N*_*i*_ = 1 to the data on accumulated annual reported HIV/AIDS cases from year 2005 to 2014, we get the estimations of transmission coefficient, modification factor of diagnosed HIV-positive individuals, diagnosis rate as *β*_*ii*_ = 0.53, ϱ = 0.11, *δ*_*i*_ = 0.14, which are listed in Table 1. Plot of data fitting is shown in Fig. 1(a), where squares denote the real data, areas from light to dark denote the 50%, 90%, 95% and 99% confidence intervals due to parameter uncertainties. The value of *R*_0_ in community *i* is estimated as 3.56 (95%CI: [3.556, 3.568]). And the MCMC plots for *R*_0_ is described in Fig. 1(b), from which we can also see that the Markov Chain has a good convergency.

### Effects of interventions on HIV infections

In this section, we numerically explore the effects of interventions, involved in implementation periods, scales of implementation of strategies and network structures on HIV infections among MSM population in China.

#### Effects of the implementation of interventions on *R*_0_

**Figure 2:**
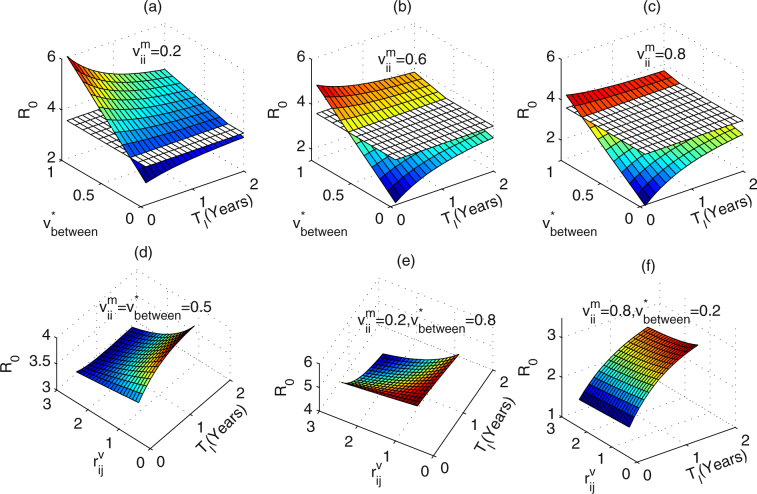
Effects of the implementation period (*T*_*l*_) of interventions on *R*_0_. The white plane denotes the value of *R*_0_ when no intervention is implemented. (**a**) ${v}_{ii}^{m}=0.2$, ${r}_{ii}^{v}={r}_{ij}^{v}=2$, (**b**) ${v}_{ii}^{m}=0.6$, ${r}_{ii}^{v}={r}_{ij}^{v}=2$, (**c**) ${v}_{ii}^{m}=0.8$, ${r}_{ii}^{v}={r}_{ij}^{v}=2$, (**d**) ${r}_{ii}^{v}=2$, ${v}_{ii}^{m}={v}_{between}^{\ast }=0.5$, (**e**) ${r}_{ii}^{v}=2$, ${v}_{ii}^{m}=\mathrm{0.2,}\,{v}_{between}^{\ast }=0.8$, (**f**) ${r}_{ii}^{v}=2$, ${v}_{ii}^{m}=\mathrm{0.8,}\,{v}_{between}^{\ast }=0.2$. Parameter values are ${\sigma }_{i}^{m}=\mathrm{0.02,}\,{r}_{i}^{\sigma }={r}_{ii}^{v}$. Other parameter values are described in Table 1.

We initially fix the number of communities covered by the interventions to be *n* = 10, and adopt the regular networks with each node connecting to the nearest *k* = 2 neighbours. To study the variation in *R*_0_ with the implementation period of interventions (*T*_*l*_) as well as the maximum within- and between-community impacts of interventions (${v}_{ii}^{m}$ and ${v}_{ij}^{m}$), here, we suppose there is no any difference between community *i* and community *j*. For simplicity, we fix the wanning rates of the impacts of interventions, and vary the mean maximum between-community impacts of interventions ${v}_{between}^{\ast }({v}_{ij}^{m}={v}_{between}^{\ast }/k)$ and the implementation period of interventions (*T*_*l*_). Figure 2(a–c) describe the results when the maximum within-community impacts of interventions are ${v}_{ii}^{m}=0.2,0.6$ and 0.8, respectively. The white plane in each figure denotes the value of *R*_0_ when no intervention is implemented. From Fig. 2(a–c) we can get that *R*_0_ increases with increasing mean maximum between-community impacts of interventions (${v}_{between}^{\ast }$) or decreasing maximum within-community impacts of interventions (${v}_{ii}^{m}$). Interestingly, frequent implementation of interventions may not always lead to *R*_0_ decline. In particular, when ${v}_{between}^{\ast }$ is below a critical value, *R*_0_ increases as the *T*_*l*_ increases, while the ${v}_{between}^{\ast }$ is greater than the critical value, *R*_0_ decreases with increasing the *T*_*l*_. What’s more, the comparison between Fig. 2(a–c) indicates that the critical value becomes larger as the maximum within-community impacts of interventions (${v}_{11}^{m}={v}_{22}^{m}$) get larger. This implies that reducing the implementation period of interventions may not always lead to the basic reproduction number decline, depending on the maximum between-community impact of interventions.

Secondly, we plot the variation in *R*_0_ with the implementation period of interventions (*T*_*l*_) as well as the wanning rates of within- and between-community impacts of interventions (${r}_{ii}^{v}$ and ${r}_{ij}^{v}$), as shown in Fig. 2(d–f). By Fig. 2(d), it is easy to get that when the ${r}_{ij}^{v}$ are below a critical value, *R*_0_ increases as the *T*_*l*_ increases. While, *R*_0_ decreases with increasing the *T*_*l*_ for relatively large ${r}_{ij}^{v}$. Figure 2(e) shows that if the ${v}_{ii}^{m}$ are relatively small while the ${v}_{between}^{\ast }$ are significantly large, the critical value becomes much larger. Yet, this critical value becomes much smaller if the ${v}_{ii}^{m}$ are relatively large while the ${v}_{between}^{\ast }$ are quite small, as shown in Fig. 2(f). This implies that whether reducing the implementation period of interventions could lead to the basic reproduction number decline not only depend on the maximum between-community impacts of interventions, but also the wanning rates of between-community impacts of interventions.

Figure 2 indicates that if the between-community impacts are small enough compared with the within-community impacts, and the wanning rate of between-community impacts of interventions are large enough, interventions should be implemented as frequently as possible in terms of getting smaller reproduction number (or less new infections). Otherwise, if the between-community impacts are relatively large compared with the within-community impacts, and the wanning rate of between-community impacts of interventions are relatively small, we could implement the interventions less frequently. Meanwhile, from Fig. 2 we can also find that enhancing within-community impacts of the interventions can effectively lead to *R*_0_ decline, and hence result in a reduction in new infections. Thus, interventions such as pamphlet distribution should be encouraged which can alert high-risk group to reduce the high risk behaviors or the contact cross communities.

#### Effects of number of communities

We examine the effects of the number of communities covered by the interventions. Here, WS (Watts and Strogatz) network and random network are adopted to illustrate the different network structure^30^. For WS network, each node is connected to the nearest *k* neighbours and each connection is rewired with probability *p*. For random network, each node is connected to others randomly with the mean number of neighbours fixed to *k*.

**Figure 3:**
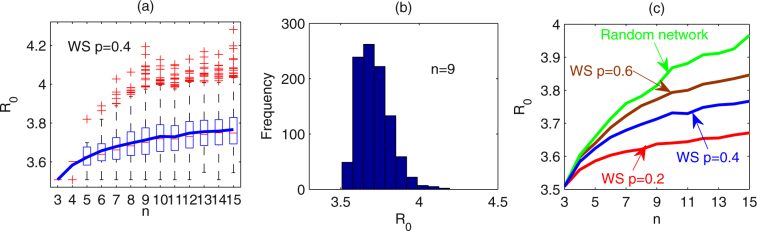
Effects of the number of communities covered by interventions on *R*_0_. The number of communities varies from 3 to 15. For each *n*, 1000 simulations are implemented. WS networks with *k* = 2, *p* = 0.2, 0.4, 0.6 and random network are used. Mean number of neighbour communities for each community is *k* = 2. (**a**) Box-plot of *R*_0_ for each *n* (WS network with *p* = 0.4). (**b**) Histogram for the value of *R*_0_ when *n* = 9 (WS network with *p* = 0.4). (**c**) Mean values of *R*_0_ for each *n* for WS networks with *p* = 0.2, 0.4, 0.6 and random network, respectively. *T*_*l*_ = 1/2, ${v}_{ii}^{m}=0.5$, ${v}_{between}^{\ast }=0.5$, ${\sigma }_{i}^{m}=\mathrm{0.02,}\,{r}_{ii}^{v}={r}_{ij}^{v}=\mathrm{2,}\,{r}_{i}^{\sigma }=2$. Other parameters are described in Table 1.

Firstly, WS networks with *k* = 2, *p* = 0.4 are chosen. Let the number of communities (*n*) covered by interventions vary from 3 to 15. For each *n*, 1000 runs are carried out and we give the box plot of *R*_0_ for each *n* (as shown in Fig. 3(a)). It follows from Fig. 3(a) that the mean value of *R*_0_ increases as the number of communities *n* increases. Specially, the histogram for the value of *R*_0_ when *n* = 9 is shown in Fig. 3(b), which gives mean of *R*_0_ as 3.7171 with standard deviation 0.1028.

Furthermore, we choose WS networks with rewired probabilities of *p* = 0.2, 0.6 and random networks with each node having a mean of *k* = 2 neighbours. With different network structures, we examine the effects of number of communities covered by the interventions on the basic reproduction number *R*_0_. For each network structure and given number of communities (*n*), 1000 runs are conducted. Figure 3(c) illustrates the mean value of basic reproduction number under each network structure, where red, blue, brown and green color curves correspond to WS networks with rewired probability of *p* = 0.2, 0.4, 0.6 and random network. It shows no matter what the network structure is the basic reproduction number increases as the number of communities increases.

**Figure 4:**
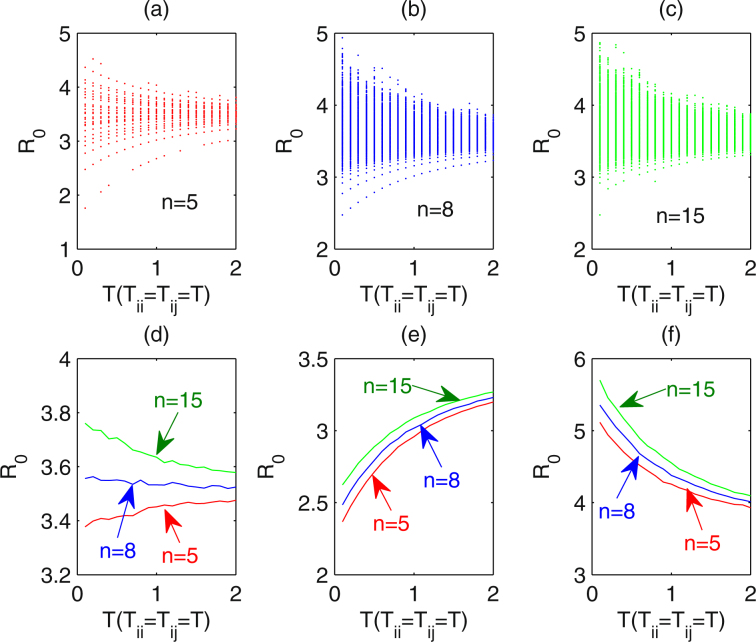
Effects of the number of communities covered by the interventions and implementation period *T*_*l*_ on *R*_0_. Random networks with each node has a mean number of 2 neighbours are adopted. The implementation period of interventions varies from 0.1 to 2 years. For each *n* and *T*_*l*_, 1000 simulations are implemented. For (**a**) to (**d**), ${v}_{ii}^{m}=0.55$, ${v}_{between}^{\ast }=0.45$. The number of communities covered by interventions is (**a**) *n* = 5, (**b**) *n* = 8, (**c**) *n* = 15. (**d**) The mean value of *R*_0_ as a function of *T*_*l*_ for each *n*. (**e**,**f**) The mean value of *R*_0_ as a function of *T*_*l*_ for *n* = 5, 8 and 10 when ${v}_{ii}^{m}=0.3$, ${v}_{between}^{\ast }=0.7$ and ${v}_{ii}^{m}=0.7$, ${v}_{between}^{\ast }=0.3$, respectively. ${\sigma }_{i}^{m}=\mathrm{0.02,}\,{r}_{ii}^{v}={r}_{ij}^{v}=\mathrm{2,}\,{r}_{i}^{\sigma }=2$. Other parameters are described in Table 1.

When the within-community impacts of interventions are only a little larger than those of between-community, for example ${v}_{ii}^{m}=\mathrm{0.55,}\,{v}_{ij}^{m}={v}_{between}^{\ast }/k,\,{v}_{between}^{\ast }=\mathrm{0.45,}\,k=2$, we study the variation in the basic reproduction number *R*_0_ with the implementation period of interventions (*T*_*l*_). We fix the number of communities covered by the interventions as *n* = 5, 8 and 15, and random networks with each node having mean number of 2 neighbours are adopted. For each *n* and *T*_*l*_, 1000 runs are carried out and variations in *R*_0_ are plotted in Fig.4(a–c), and the mean values of *R*_0_ for various *n* are shown in Fig. 4(d). Figure 4(a–c) show that the variation range of *R*_0_ shrinks as the period *T*_*l*_ increases. The comparison between (a), (b) and (c) indicates that the variation range of *R*_0_ becomes larger as the number of communities *n* increases. It is worth stressing that for *n* = 5 the mean value of *R*_0_ increases with increasing the implementation period *T*_*l*_, however, the mean value of *R*_0_ decreases with the period *T*_*l*_ for *n* = 15 (as shown in Fig. 4(d)). This implies that, in such scenario, in which the within-community impacts of interventions are only a little larger than the between-community impacts, when the number of communities covered by the interventions is relatively small, interventions should be implemented as frequently as possible, however, when the number of communities covered by interventions is relatively large, frequency of interventions should be reduced, in terms of inducing less new infections (i.e. smaller *R*_0_). A repeat of Fig. 4(d) for ${v}_{ii}^{m}=\mathrm{0.7,}\,{v}_{between}^{\ast }=0.3$ (the within-community impacts of interventions are much larger than the between-community impacts), the mean value of *R*_0_ increases with the period *T*_*l*_ no matter of the number of communities, as shown in Fig. 4(e). Conversely, if the within-community impacts of interventions are smaller than the between-community impacts, the mean value of *R*_0_ decreases with the period *T*_*l*_ no matter of the number of communities, as shown in Fig. 4(f). From Figs 3 and 4 we can get that the more communities the interventions cover, the larger the *R*_0_ and consequently the more new infections. Hence, this type of intervention is harmful for disease control. However, whether frequently implementing interventions reduces the *R*_0_ depends on the number of communities that interventions cover and relative value of within- and between-community impacts of interventions.

#### Effects of the structure of networks

In the following, we study the effects of the structure of networks on the disease transmission. From Fig. 3(c) we can obtain that as the network structure changes from WS network with rewired probability of *p* = 0.2, 0.4 to *p* = 0.6, and then to random network, the basic reproduction number quickly increases as the number of communities increases. For a given number of communities covered by the interventions, the WS network with greater rewired probability induces more new infections, and moreover, random network induces the greatest new infections, compared to WS networks. This indicates that if communities connected randomly, implementing interventions will lead to more new infections.

**Figure 5:**
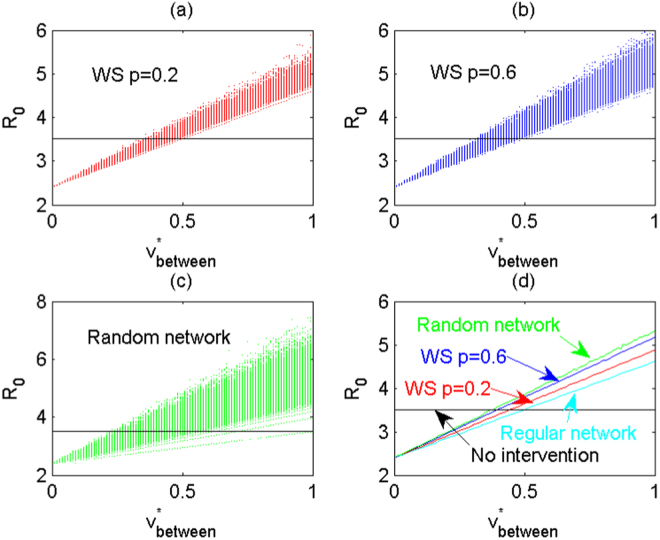
With different network structures the effects of the maximum between-community impacts of interventions on *R*_0_. The number of communities covered by interventions is *n* = 10 and each community has a mean of *k* = 2 neighbours. The maximum within-community impacts of interventions are fixed at ${v}_{ii}^{m}=0.5$, and the maximum between-community impacts ${v}_{between}^{\ast }$ vary from 0 to 1. For each network structure and each ${v}_{between}^{\ast }$, 1000 simulations are implemented. (**a**) WS network with rewired probability of *p* = 0.2. (**b**) WS network with rewired probability of *p* = 0.6. (**c**) Random network. (**d**) Mean value of *R*_0_ for WS network with rewired probability of *p* = 0.2, 0.6 and random network, respectively. Here, *T*_*l*_ = 1/2, ${\sigma }_{i}^{m}=\mathrm{0.02,}\,{r}_{ii}^{v}={r}_{ij}^{v}=\mathrm{2,}\,{r}_{i}^{\sigma }=2$. Other parameters are described in Table 1.

Suppose the number of communities covered by the interventions is *n* = 10, and each node has a mean number of *k* = 2 neighbours. With different network structures, we examine the effects of maximum between-community impacts of interventions (${v}_{ij}^{m}={v}_{between}^{\ast }$) on *R*_0_. Again, 1000 runs for WS network with rewire probability of *p* = 0.2, 0.6 and random network are carried out. We calculate the variation of *R*_0_, shown in Fig. 5(a–c), respectively, and Fig. 5(d) gives the mean value of *R*_0_ for each network structure. Figure 5 shows *R*_0_ increases as ${v}_{ij}^{m}$ increases, and greater rewiring probability in WS network induces greater reproduction number with large variation. Furthermore, mean value and the variation of *R*_0_ are the largest for random network (shown in Fig. 5(d)). This implies that on average stronger randomness of the network will lead to a larger *R*_0_ and hence more new infections. Similarly, we can examine the effects of the network structure on the number of HIV/AIDS cases, that is, strong randomness of the network structure results in a large mean value of the number of HIV/AIDS cases with great variation at given time (see detailed Figure S1 in SI). We then can conclude that strong randomness of the network will lead to larger *R*_0_ and more HIV/AIDS cases, This indicates that intervention manners are essential and should be chosen carefully, and in particular, interventions with low random contacts for individuals from different communities should be encouraged.

## Conclusions and Discussions

Intervention measures are designed to decrease the frequency of high risk behaviors within communities, but they may inevitably change the connection network among communities and consequently make the isolated communities become connected to each other, and thus potentially increase the frequency of high risk behaviors between communities. A multi-community model is formulated to study the two-side effects of intervention measures on HIV transmission dynamics. In this model, communities are connected according to the adjacent matrix which is determined by the network structure. Interventions are supposed to be implemented periodically, and the impacts of interventions are modelled dynamically. Based on the the number of annual reported HIV/AIDS cases who are infected through homosexual behaviors from the year 2005 to 2014 in China, MCMC method is carried out to estimate the unknown parameters involved in single community model (1). The basic reproduction number for a single community is estimated as 3.56 (95%CI [3.556, 3.568]), which is in the ranges of estimations for Western Germany (3.43–4.08) and UK (3.38–3.96)^48^ and is associated with the estimation by Lou *et al*.^49^ and Sun *et al*.^39^.

Interesting, the impacts of interventions are modelled dynamically rather than assuming to be constants in each implementation period of interventions. By considering the wanning of impact of intervention measures, our model is much more realistic, and we can study the effect of implementation period of interventions. We found that frequent implementation of interventions may lead to the *R*_0_ decline or increase, depending on the relative level of between- and within-community impacts. In particular, if the between-community impacts of interventions are relatively small, the interventions should be implemented as frequently as possible, otherwise, the interventions should be implemented less frequently. This indicates that interventions should be implemented with much care. Interventions with low contacts for individuals from different communities, such as pamphlet distribution, media propaganda should be advocated, while interventions such as workshop, propaganda education may not be encouraged since such interventions could inevitably increase the chance for people to know new friends, which may consequently increase the frequency high-risk behaviors among communities.

We also investigated the effects of number of communities and the network structure among communities on the disease transmission. Given individuals have on average high risk behaviors with others in different communities with a fixed probability (no matter what the number of communities is), numerical studies suggest that the basic reproduction number for the whole system increases with increasing the number of communities. Thus, interventions, if implemented, should be implemented with number of communities involved as small as possible. Meanwhile, network structure does significantly affect the basic reproduction number and prevalence for the whole system. In particular, the more randomness the networks the larger the basic reproduction number, and the more HIV/AIDS cases, which in accordance with the well-know conclusion^50^ that epidemic spreads faster in random networks than in small-world networks. This further indicates that the implementation manner of interventions should be chosen carefully. Interventions with less communities involved and less random contacts between individuals should be encouraged.

The modelling approach developed here allows us to study the effects of the intervention measures on HIV infections among MSMs in China. Our main conclusion indicates that intervention measures may accelerate the disease transmission, depending on relative level of between- and within-community impacts, and the frequency of implementation of interventions. Then, our work shows the potential shortcomings of the certain interventions on the basis of mathematical models, and may induce more new HIV infections if not implemented properly. The findings can help to guide the policy maker to choose the appropriate intervention measures, and to implement the interventions with proper frequency.

## Electronic supplementary material

Supplementary Information
