# Supplementary material for: Frequent implementation of interventions may increase HIV infections among MSM in China
Source: Sci Rep. 2018 Jan 11;8:451. doi: 10.1038/s41598-017-18743-7 (PMC5765000; doi:10.1038/s41598-017-18743-7)
Supplement: Supplementary file 1 — Supplementary Information [file 41598_2017_18743_MOESM1_ESM.pdf]

Frequent implementation of interventions may increase HIV infections among MSM in China

Xiaodan Sun, Yanni Xiao, [1] Zhihang peng, Ning Wang

1 Existence of the periodic disease-free equilibrium

We can show that system (2)-(3) has a disease-free periodic solution.

Lemma A.1 *System (2)-(3) has a T_l−periodic solution* $\hat{S}(t) = (\hat{S}_1(t), 0, 0, 0, \cdots, \hat{S}_i(t), 0, 0, 0, \cdots, \hat{S}_n(t), 0, 0, 0)$.

Proof To show the existence of disease-free periodic solution of system (2)-(3), we consider the following disease-free subsystem.

$$\begin{cases} \frac{dS_i}{dt} &= U_i - (\mu_i + \sigma_i(t) + d)S_i, \\ \frac{d\sigma_i(t)}{dt} &= -r_i^{\sigma}\sigma_i(t), \quad t \neq kT_l, \\ \sigma_i(T_l^+) &= \sigma_i^m, \quad\quad t = kT_l. \end{cases} \tag{S.1}$$

Let $S(t) = (S_1(t), S_2(t), \cdots, S_n(t))$, $U = (U_1, U_2, \cdots, U_n)$, $\mu = (\mu_1, \mu_2, \cdots, \mu_n)$ and $\sigma(t) = (\sigma_1(t), \sigma_2(t), \cdots, \sigma_n(t))$, where $\sigma_i(t) = \sigma_i^0 e^{-r_i^{\sigma}(t-kT_l)}$, $k = 1, 2, \cdots$, $kT_l \le t < (k+1)T_l$, we have

$$\frac{dS(t)}{dt} = U - (\mu + \sigma(t) + d)S(t), \quad t \neq kT_l. \tag{S.2}$$

Clearly, (S.2) has a unique positive T_l periodic solution

$$\hat{S}(t) = e^{-\int_0^t (\mu+\sigma(s)+d)ds}\left(\hat{S}(0) + U\int_0^t e^{\int_0^s(\mu+\sigma(\xi)+d)d\xi}ds\right).$$

which is globally attractive in R_+^n, where

$$\hat{S}(0) = \frac{U\int_0^{T_l} e^{\int_0^s(\mu+\sigma(\xi)+d)d\xi}ds}{e^{\int_0^{T_l}(\mu+\sigma(s)+d)ds} - 1}.$$

Thus, system (2)-(3) has a unique disease free periodic solution $x^*(t) = (\hat{S}_1(t), 0, 0, 0, \cdots, \hat{S}_i(t), 0, 0, 0, \cdots, \hat{S}_n(t), 0, 0, 0)$.

[1]Corresponding author. E-mail: yxiao@mail.xjtu.edu.cn

2 The basic reproduction number

In the following, we define the basic reproduction number of (2)-(3) by using the theory proposed by Wang and Zhao.[1] System (2)-(3) is equivalent to the following system

$$\frac{d}{dt}x(t) = \mathcal{F}(t,x) - \mathcal{V}(t,x). \tag{S.3}$$

where $x = (I_1, D_{I1}, D_{A1}, \cdots, I_i, D_{Ii}, D_{Ai}, \cdots, I_n, D_{In}, D_{An}, S_1, \cdots, S_i, \cdots, S_n)$,

$$\mathcal{F}(t,x) = \begin{pmatrix} B_1(t) \\ 0 \\ 0 \\ \vdots \\ B_n(t) \\ 0 \\ 0 \\ 0 \\ \vdots \\ 0 \end{pmatrix}, \mathcal{V}(t,x) = \begin{pmatrix} (d+\delta_1)I_1 \\ -\rho\delta_1 I_1 + (\xi + d + \alpha_I) \\ -(1-\rho)\delta_1 I_1 - \xi D_{I1} + (d+\alpha_A)D_{A1} \\ \vdots \\ (d+\delta_n)I_n \\ -\rho\delta_n I_n + (\xi + d + \alpha_I) \\ -(1-\rho)\delta_n I_n - \xi D_{In} + (d+\alpha_A)D_{An} \\ -U_1 + B_1(t) + (\mu_1(t) + d)S_1 \\ \vdots \\ -U_n + B_n(t) + (\mu_n(t)+d)S_1 \end{pmatrix},$$

with $B_i(t) = \beta_{ii}(1 - v_{ii}(t))S_i\frac{I_i + \varrho D_{Ii} + \epsilon D_{Ai}}{N_i} + \sum_{j\neq i}^{n} \beta_{ij} m_{ij} v_{ij}(t) S_i \frac{I_j + \varrho D_{Ij} + \epsilon D_{Aj}}{N_j}$.

It is obvious that conditions (A1)-(A5) in reference[1] are satisfied. Let $f(t, x(t)) = \mathcal{F}(t,x) - \mathcal{V}(t,x)$ and $\tilde{M}(t) = (\frac{\partial f_i(t,x^*(t))}{\partial x_j})_{3n+1\leq i,j\leq 4n}$, where $x^*(t) = (0,0,0,\cdots,0,0,0,S_1^*,\cdots, S_i^*,\cdots,S_n^*)$ is the disease-free periodic solution and x_i is the $i-$th component of $f(t,x(t))$ respectively. Then we have

$$\tilde{M}(t) = \begin{pmatrix} -(\mu_1 + \sigma_1(t) + d) & 0 & \cdots & 0 \\ 0 & -(\mu_2 + \sigma_2(t) + d) & \cdots & 0 \\ \vdots & \vdots & \ddots & \vdots \\ 0 & 0 & \cdots & -(\mu_n + \sigma_n(t) + d) \end{pmatrix},$$

and it is easy to obtain that $r(\Phi_{\tilde{M}}(\omega)) < 1$, where $\Phi_M(t)$ is the mondromy matrix of the linear T_l- period system $\frac{dy}{dt} = \tilde{M}(t)y$ and $r(\Phi_{\tilde{M}}(\omega))$ is the spectral radius of $\Phi_{\tilde{M}}(\omega)$. Thus, the condition (A6) in reference[1] also holds.

Let $F(t) = (\frac{\partial \mathcal{F}_i(t,x^*(t))}{\partial x_j})_{1\leq i,j\leq 3n}$ and $V(t) = (\frac{\partial \mathcal{V}_i(t,x^*(t))}{\partial x_j})_{1\leq i,j\leq 3n}$, where $\mathcal{F}_i(t,x)$ and $\mathcal{V}_i(t,x)$ are

the $i-$th component of $\mathcal{F}(t,x)$ and $\mathcal{V}(t,x)$, respectively. Then, we have

$$F(t)=\begin{pmatrix} A_{11}(t) & A_{11}(t)\varrho & A_{11}(t)\epsilon & \cdots & A_{1n}(t) & A_{1n}(t)\varrho & A_{1n}(t)\epsilon \\ 0 & 0 & 0 & \cdots & 0 & 0 & 0 \\ 0 & 0 & 0 & \cdots & 0 & 0 & 0 \\ \vdots & \vdots & \vdots & \ddots & \vdots & \vdots & \vdots \\ A_{n1}(t) & A_{n1}(t)\varrho & A_{n1}(t)\epsilon & \cdots & A_{nn}(t) & A_{nn}(t)\varrho & A_{nn}(t)\epsilon \\ 0 & 0 & 0 & \cdots & 0 & 0 & 0 \\ 0 & 0 & 0 & \cdots & 0 & 0 & 0 \end{pmatrix},$$

and

$$V=\begin{pmatrix} d+\delta_1 & 0 & 0 & \cdots & 0 & 0 & 0 \\ -\rho\delta_1 & \xi+d+\alpha_I & 0 & \cdots & 0 & 0 & 0 \\ -(1-\rho)\delta_1 & -\xi & d+\alpha_A & \cdots & 0 & 0 & 0 \\ \cdots & \cdots & \cdots & \ddots & \cdots & \cdots & \cdots \\ 0 & 0 & 0 & \cdots & d+\delta_n & 0 & 0 \\ 0 & 0 & 0 & \cdots & -\rho\delta_n & \xi+d+\alpha_I & 0 \\ 0 & 0 & 0 & \cdots & -(1-\rho)\delta_n & -\xi & d+\alpha_A \end{pmatrix},$$

where $A_{ii}=\beta_{ii}(1-v_{ii}(t)), i=1,\cdots,n$, $A_{ij}=\beta_{ij}m_{ij}v_{ij}(t)\frac{\hat{N}_i(t)}{\hat{N}_j(t)}, i,j=1,\cdots,n$, and $\hat{N}_i(t)=\frac{U_i}{\mu_i+\sigma_i(t)+d}$.

Let $Y(t,x)$ be a $3n\times 3n$ matrix solution of the following system.

$$\frac{dY(t,s)}{dt}=-V(t)Y(t,s), \text{for any} \quad t\geq s, Y(s,s)=I,$$

where I is a $3n\times 3n$ identity matrix. Therefore, the condition (A7) in reference[1] holds.

Define $\psi(t)$ as the initial periodic distribution of infected individuals with periodic T_l. Then, the distribution of infected individuals infected at time s and are still infected individuals at time t can be given by $Y(t,s)F(s)\psi(t)$. Let C_{T_l} be the ordered Banach space of all T_l- periodic functions from R to R^{3n}, which is equipped with the maximum norm $||\cdot||$ and the positive cone $C^+_{T_l}:=\{\psi\in C_{T_l}:\psi(t)\geq 0,\forall t\in \mathrm{R}\}$. A linear operator $L: C_{T_l}\to C_{T_l}$ is defined as follows.

$$(L\psi)(t)=\int_0^\infty Y(t,t-a)F(t-a)\psi(t-a)da, \forall t\in \mathrm{R}, \psi\in C_{T_l}.$$

Then, we can define the basic reproduction number as

$$R_0:=\rho(L).$$

From Wang and Zhao,[1] we have the following Lemma.

Lemma S1(See[1]) The following statements are valid:

- If $r(W(T_l, \lambda)) = 1$ has a positive solution λ_0, then λ_0 is an eigenvalue of L, and hence $R_0 > 0$.

- If $R_0 > 0$, then $\lambda = R_0$ is the unique solution of $r(W(T_l, \lambda)) = 1$.

- $R_0 = 0$ if and only if $r(W(T_l, \lambda)) < 1$ for all $\lambda > 0$.

On the basis of this Lemma, we can calculate the basic reproduction number numerically by finding the positive solution λ_0 of $r(W(T_l, \lambda)) = 1$.

3 Effects of interventions on HIV infections

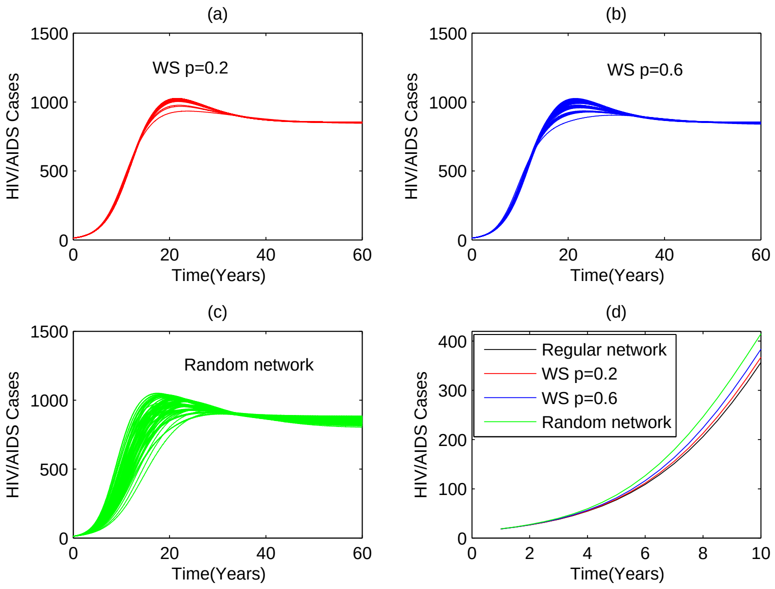

Figure S1: **Effects of the network structures on the number of HIV/AIDS cases of each community.** The number of communities is $n = 10$ and each community has a mean of $k = 2$ neighbours. The maximum within-community impacts of interventions are $v_{ii}^m = 0.5$, and the maximum between-community impacts are $v_{between}^* = 0.5$. For each network structure 100 simulations are conducted. (a) WS network with rewired probability of $p = 0.2$. (b) WS network with rewired probability of $p = 0.6$. (c) Random network. Here, $T_l = 1/2$, $\sigma_i^m = 0.02$, $r_{ii}^v = r_{ij}^v = 2$, $r_i^\sigma = 2$. Other parameters are described in Table 1 in the main text.

References

1. Wang, W. & Zhao, X. Q. Threshold dynamics for compartmental epidemic models in periodic environments. J. Dynam. Diff. Equ. **20**, 699-717 (2008).
